# Microglia and macrophage exhibit attenuated inflammatory response and ferroptosis resistance after RSL3 stimulation via increasing Nrf2 expression

**DOI:** 10.1186/s12974-021-02231-x

**Published:** 2021-10-30

**Authors:** Yu Cui, Zhaolong Zhang, Xin Zhou, Zhiyuan Zhao, Rui Zhao, Xiangyu Xu, Xiangyi Kong, Jinyang Ren, Xujin Yao, Qian Wen, Feifei Guo, Shengli Gao, Jiangdong Sun, Qi Wan

**Affiliations:** 1 Institute of Neuroregeneration & Neurorehabilitation, Department of Pathophysiology, Qingdao University, Ningxia Road 308, Qingdao, 266071 China; 2 The Affiliated Hospital of Qingdao University, Jiangsu Road 16, Qingdao, 266000 Shandong China; 3 Department of Biomedical Center, Qingdao University, Qingdao, 266071 China; 4 School of Basic Medicine, Qingdao University, Ningxia Road 308, Qingdao, 266071 China

**Keywords:** Ferroptosis, RSL3, Neuroinflammation, Microglia, Macrophage, LPS

## Abstract

**Background:**

Many neurological diseases involve neuroinflammation, during which overproduction of cytokines by immune cells, especially microglia, can aggregate neuronal death. Ferroptosis is a recently discovered cell metabolism-related form of cell death and RSL3 is a well-known inducer of cell ferroptosis. Here, we aimed to investigate the effects of RSL3 in neuroinflammation and sensitivity of different type of microglia and macrophage to ferroptosis.

**Methods:**

Here, we used quantitative RT-PCR analysis and ELISA analysis to analyze the production of proinflammatory cytokine production of microglia and macrophages after lipopolysaccharides (LPS) stimulation. We used CCK8, LDH, and flow cytometry analysis to evaluate the sensitivity of different microglia and macrophages to RSL3-induced ferroptosis. Western blot was used to test the activation of inflammatory signaling pathway and knockdown efficiency. SiRNA-mediated interference was conducted to knockdown GPX4 or Nrf2 in BV2 microglia. Intraperitoneal injection of LPS was performed to evaluate systemic inflammation and neuroinflammation severity in in vivo conditions.

**Results:**

We found that ferroptosis inducer RSL3 inhibited lipopolysaccharides (LPS)-induced inflammation of microglia and peritoneal macrophages (PMs) in a cell ferroptosis-independent manner, whereas cell ferroptosis-conditioned medium significantly triggered inflammation of microglia and PMs. Different type of microglia and macrophages showed varied sensitivity to RSL3-induced ferroptosis. Mechanistically, RSL3 induced Nrf2 protein expression to inhibit RNA Polymerase II recruitment to transcription start site of proinflammatory cytokine genes to repress cytokine transcription, and protect cells from ferroptosis. Furthermore, simultaneously injection of RSL3 and Fer-1 ameliorated LPS-induced neuroinflammation in in vivo conditions.

**Conclusions:**

These data revealed the proinflammatory role of ferroptosis in microglia and macrophages, identified RSL3 as a novel inhibitor of LPS-induced inflammation, and uncovered the molecular regulation of microglia and macrophage sensitivity to ferroptosis. Thus, targeting ferroptosis in diseases by using RSL3 should consider both the pro-ferroptosis effect and the anti-inflammation effect to achieve optimal outcome.

**Supplementary Information:**

The online version contains supplementary material available at 10.1186/s12974-021-02231-x.

## Background

Inflammation is a protective response by the body to remove detrimental stimuli, as well as an initiation of the healing process for the damaged tissue. The innate immune response mainly contributes to acute inflammation triggered by microbial infection or tissue damage [1, 2]. Pattern recognition receptors (PRRs) on innate immune cells are established to be responsible for sensing the presence of microorganisms by recognizing pathogen-associated molecular patterns (PAMPs). Toll-like receptors (TLRs) are the main PRRs which can recognize conserved microbial components such as bacterial lipopolysaccharide (LPS) and dsRNA, and activate downstream signaling cascades to induce proinflammatory cytokine production to attack pathogens [3, 4]. Many neurological diseases such as multiple sclerosis, ischemic stroke, and Parkinson’s disease involve neuroinflammation, during which overproduction of cytokines by immune cells, namely a cytokine storm, can further aggregate neuronal death [5]. Thus, negative regulation of TLR signaling to attenuate inflammation is important for protecting the host from excessive inflammatory immune responses and maintaining immune homeostasis [6, 7].

Ferroptosis is defined as a metabolism-regulated cell death, which is characterized by the iron-dependent accumulation of lipid hydroperoxides to lethal levels [8, 9]. Many types of inducers are capable to trigger ferroptosis. RSL3 serves as a GPX4 inhibitor to increase lipid peroxidation [10]. System xc^-^ inhibitors such as erastin and its analogs trigger ferroptosis by preventing cystine import and causing GSH depletion [11, 12]. In addition to ferroptosis inducers, a variety of pharmacological inhibitors of ferroptosis have also been discovered. Inhibitors of lipid peroxidation, such as Ferrostatin-1 (Fer-1) and liproxstatins, suppress ferroptosis [13]. Inhibitors of iron metabolism and iron chelators, such as deferoxamine and ciclopirox, suppress ferroptosis by reducing the availability of iron [11]. Till now, various ways of cell death including apoptosis, necrosis, necroptosis, and pyroptosis contribute to inflammation by releasing intracellular or extracellular substances [14–16]. However, the regulation of ferroptosis and its inducers in inflammation especially in neuroinflammation is not fully understood.

At present, ferroptosis has been revealed to participate in many diseases, such as cancer, ischemia-reperfusion injury and neurodegeneration, and inhibiting ferroptosis has been raised to treat some diseases, particularly cancer [9, 17–19]. However, some cancer cells exhibit ferroptosis resistance which hinder the treatment effects [20]. Notably, accumulating data reveal the molecular mechanism of ferroptosis resistance. Generally, the regulatory resistant pathways of ferroptosis involve iron metabolism [21, 22], lipid metabolism [23, 24], and anti-oxidant signaling regulation [25, 26]. Although the vulnerability of ferroptosis has been documented in many types of cells, reports of the ferroptotic sensitivity and regulatory mechanism of different innate immune cells, especially microglia and macrophages are still limited [27], and need further investigation.

In this study, we showed for the first time that ferroptosis inducer RSL3 inhibits inflammation of microglial cells and peritoneal macrophages (PMs) upon LPS stimulation, whereas cell ferroptosis-conditioned medium triggered inflammation obviously. Further analysis showed that BV2 cells and PMs were resistant to ferroptosis, although bone marrow-derived macrophages (BMDMs) and RAW264.7 cells were very sensitive to RSL3-induced ferroptosis. In addition, BV2 cells and PMs exhibited suppressed inflammation by increasing Nrf2 protein abundance. Treatment with RSL3 and Fer-1 reduced systemic inflammation in in vivo conditions.

## Methods

### Mice

Six- to 10-week-old, sex-matched mice C57BL/6 mice were used for experiments in this paper unless otherwise indicated. All mice were bred in specific pathogen-free conditions. All animal experiments were conducted in compliance with National Institutes of Health Guidelines and were approved by the institutional animal care and use committee of the Qingdao University.

### Antibodies and reagents

The following antibodies from Cell Signaling Technology (CST) were used: Phospho-NF-kappa-B p65 (Ser536) Antibody (3031S), NF-kappa-B p65 (C22B4) Rabbit mAb (4764S), Phospho-SAPK/JNK (Thr183/Tyr185) (81E11) Rabbit mAb (4668), SAPK/JNK Antibody (9252), p38 MAPK (D13E1) XP® Rabbit mAb (8690), Phospho-p38 MAPK (Thr180/Tyr182) (12F8) Rabbit mAb (4631), Phospho-p44/42 MAPK (Erk1/2) (Thr202/Tyr204) Antibody (9101S), and p44/42 MAPK (Erk1/2) Antibody (4695). Nrf2 antibody was purchased from Abcam (ab137550), RNA Polymerase II antibody was purchased from Sigma (05-623), and β-action was purchased from Proteintech (60008-1-Ig). RSL3 (S8155) was from Selleck, Ferrostatin-1 (SML0583) was from Sigma, and C11-BODIPY 581/591 lipid peroxidation sensor (D3861) was from Life Technologies. LPS (LPS Ultrapure, *Escherichia coli* 0111: B4) was from Sigma. PEG300 and Tween 80 were from Selleck.

### Establishment of systemic LPS injection model

For the establishment of LPS-induced inflammation model, C57BL/6 mice received a single intraperitoneal (i.p.) injection of 10 or 5 mg/kg LPS in PBS or PBS alone as described [28, 29]. TNFα, IL-6, and IL1-β were measured by ELISA in serum of mice given intraperitoneal injection of LPS (10 mg/kg). Lungs were collected for HE staining. Brain tissues were used for flow cytometry analysis or qRT-PCR analysis (5 mg/kg). The animals were divided into three experimental groups in each experiment: group 1, treated with PBS without LPS; group 2, treated with LPS and vehicle; group 3, treated with LPS, RSL3 plus Fer-1. The RSL3 (5 mg/kg body weight, dissolved in 2% DMSO+30% PEG300+2%Tween 80+H_2_O) plus Fer-1(5 mg/kg body weight, dissolved in 2% DMSO+40% PEG300+2%Tween80+H_2_O) or corresponding vehicle was administered i.p. daily for 2 days. LPS was administered on day 2 for a single challenge.

### Histology staining

Perfused lungs from anesthetized mice were dissected, fixed in 4% (v/v) buffered paraffin overnight. Five-microgram tissue sections were stained with hematoxylin and eosin as previously described [30]. All images were acquired with an Olympus IX53 microscope.

### Primary neonatal microglia culture

Primary neonatal microglia culture was adapted from previous report [19]. Briefly, the neonatal brain from 1 to 2 days mice were trypsinized and dissociated and cells were plated in a six-well plate in DMEM/Ham’s F12 medium containing 10% FBS, penicillin, and streptomycin. Culture media were changed every three days. Cells were allowed to reach 90% confluence. To harvest microglia, at day 14 in vitro, cultures were mildly trypsinized with trypsin solution (0.05% trypsin in DMEM/Ham’s F12) at 37 °C for 40 min. Floating cells were removed and the resulting enriched microglial cultures were trypsinized with 0.25% trypsin and plated for future experiments.

### Isolation of adult microglia

Isolation of microglia was adapted from previous report [19]. Briefly, mice were killed and brain tissue were immediately removed. Brains were minced and enzymatically dissociated with 0.5 mg/ml collagenase type III (Worthington Biochemical), 1 mg/ml dispase II (Roche Applied Science), and 1 mg/ml DNase I (sigma) in RPMI-1640 for 30 min at 37 °C. The digested tissue was centrifuged and resuspended in 30% Percoll layered on 70% Percoll. The Percoll gradient was centrifuged at 2000 rpm for 20 min h at 4 °C. Cells were collected from the 70 to 30% interface and washed with PBS. Isolated cells were then stained with fluorochrome-conjugated antibody to CD45 and CD11b and were used for further analysis.

### CCK8 assay

Cell survival was assayed by Cell Counting Kit-8 (Solarbio) based on the manufacturer’s instructions. PMs and BV2 microglia were plated in 96-well plates. After indicated treatment, CCK-8 solution was added into each well, followed by incubation for indicated times. Cell viability was determined by measuring the OD at 450 nm [31].

### Lactate dehydrogenase (LDH) release assay

LDH release was measured according to the manufacturer’s instructions (Beyotime, China). The levels of LDH were measured by analyzing LDH released in the cell culture medium. Absorbance data were obtained using a 96-well plate reader (Molecular Devices, USA) at 490 nm. LDH release (%) was calculated by calculating the ratio of experimental LDH release to control LDH release according to the manufacturer’s instructions [32].

### Immunoblot analysis

Cells were lysed and incubated at 100 °C for 15 min. Proteins were separated by SDS-PAGE and transferred to PVDF membranes (Millipore). Membranes were blocked with 5% milk in Tris buffered saline for 1 h at room temperature (RT). Membranes were then incubated with primary antibodies overnight and secondary antibodies for 1 h at RT. Membranes were then visualized by immunoblot analysis with the chemiluminescence detection system (Protein Simple) [31, 33].

### Quantitative real-time PCR (qRT-PCR) analysis

Total RNA of cultured cells or tissue was extracted with RNAfast200 purification kit (Fastagen), and reverse-transcribed with the ReverTra Ace® qPCR RT Master Mix with gDNA Remover (TOYOBO, FSQ-301). Real-time PCR was performed on the ABI Q3 machine (Thermo) with 2×RealStar Green Power Mixture (GenStar). GAPDH was used as the internal control [31, 33]. The sequences of qRT-PCR primers for the genes examined are listed in the supplementary information (Supplementary Table 1).

### Enzyme-linked immunosorbent assay (ELISA)

Secreted cytokines in cell culture supernatants or mice serum were analyzed using mouse TNFα, IL-6, and IL-1β (Absin Bioscience) ELISA kits according to the manufacturer’s instructions as previously described [31].

### Assessment of lipid peroxidation with C11-BODIPY and flow cytometry

Lipid peroxidation was tested as previously described [19]. Cells were incubated with C11-BODIPY (1 μM) for 1 h at 37 °C in cell cultures. After incubation, cells were harvested and washed with PBS plus 0.1% BSA and then resuspended in PBS plus 0.1% BSA. Cell fluorescence was acquired on a Beckman flow cytometer and analyzed with FlowJo software.

#### Measurement of intracellular ROS

The intracellular ROS levels were detected using a peroxide-sensitive fluorescent probe (DCFH-DA; Beyotime) according to the instructions of the manufacturer. Briefly, DCFH-DA was diluted at a final concentration of 10 μM and then incubated with BV2, PC12, or PMs for 30 min at 37 °C. Later, the cells were harvested and washed with PBS for twice and then cell fluorescence was acquired on a BECKMAN CytoFLEX S flow cytometer and analyzed with FlowJo software.

#### Measurement of malondialdehyde (MDA) level

The MDA levels were detected according to the instructions of the manufacturer (MDA assay kit; Beyotime). Cells were first treated with different doses of RSL3 and then were lysed with lysis buffer. The supernatant of cell lysis and standard samples were mixed with MDA working solution and boiled for 15 min at 100 °C. Later, absorbance data of the cooled supernatant was tested with a 96-well plate reader (Molecular Devices, USA) at 490 nm and 532 nM. MDA concentration was calculated as μmol/mg protein.

### Cell culture and siRNA-mediated Interference

RAW264.7 cells was a gift from Dr. Xuetao Cao (Nankai University) from American Type Culture Collection (ATCC) and cultured in endotoxin-free Dulbecco’s modified Eagle’s medium (DMEM) containing 10% fetal bovine serum (FBS, Gibco). PC12 cells and BV2 cells were obtained from ATCC and cultured in DMEM containing 10% fetal bovine serum (FBS, Gibco). Thioglycollate-elicited mouse peritoneal macrophages were prepared and cultured in DMEM medium with 10% FCS (Invitrogen). Mouse BMDMs were cultured in DMEM medium with 10% FBS and recombinant 50 ng/ml of mouse macrophage colony-stimulating factors (M-CSF; Peprotech) as previously described [34].

To silence gene expression, 20 nM siRNA was transfected into the indicated cells using standard procedures with Lipofectamine RNAiMAX Transfection Reagent (Thermo Fisher Scientific) according to the manufacturer’s instructions. Cells were stimulated and harvested for further analysis 48 h after transfection. The following siRNA sequences were used: mouse Nrf2 sense, 5′-CAGGCUAUCUCCUAGUUCU-3′; mouse Nrf2 anti-sense, 5′-AGAACUAGGAGAUAGCCUG-3′ (from RIBOBIO); mouse GPX4 sense, 5′-GAUGAAUUAUGUUCAGAAAtt-3′; and mouse GPX4 anti-sense, 5′-UUUCUGAACAUAAUUCAUCtt-3′.

### Chromatin immunoprecipitation assay (CHIP)

CHIP assay was performed according to the previous report [33]. Briefly, PMs or Bv2 cells were cross-linked for 30 min on ice with 2% formaldehyde and lysed for 10 min on ice. The lysates were then sonicated to obtain DNA fragments of an average length of 500–1000 bp. The fragmented lysates were subjected to immunoprecipitation with the indicated Abs (IgG or RNA Pol II antibody). The recovered DNA was used as templates for qRT-PCR analysis. The primers used for qRT-PCR were listed in the supplementary information (Supplementary Table 2).

### Statistical analysis

The statistical analysis was performed using GraphPad Prism software. All experiments were performed for three or more times unless otherwise indicated. All data were shown as means ± SD. To compare the statistical significance of two independent groups, Student’s *t* test was used. Differences between the groups were determined using One-way ANOVA analysis followed by Tukey test or Dunnett test, or two-way-ANOVA analysis followed by Bonferroni post hoc test.

## Results

### Ferroptosis inducer RSL3 inhibits LPS-induced inflammatory cytokine production in PMs and microglia

**Fig. 1:**
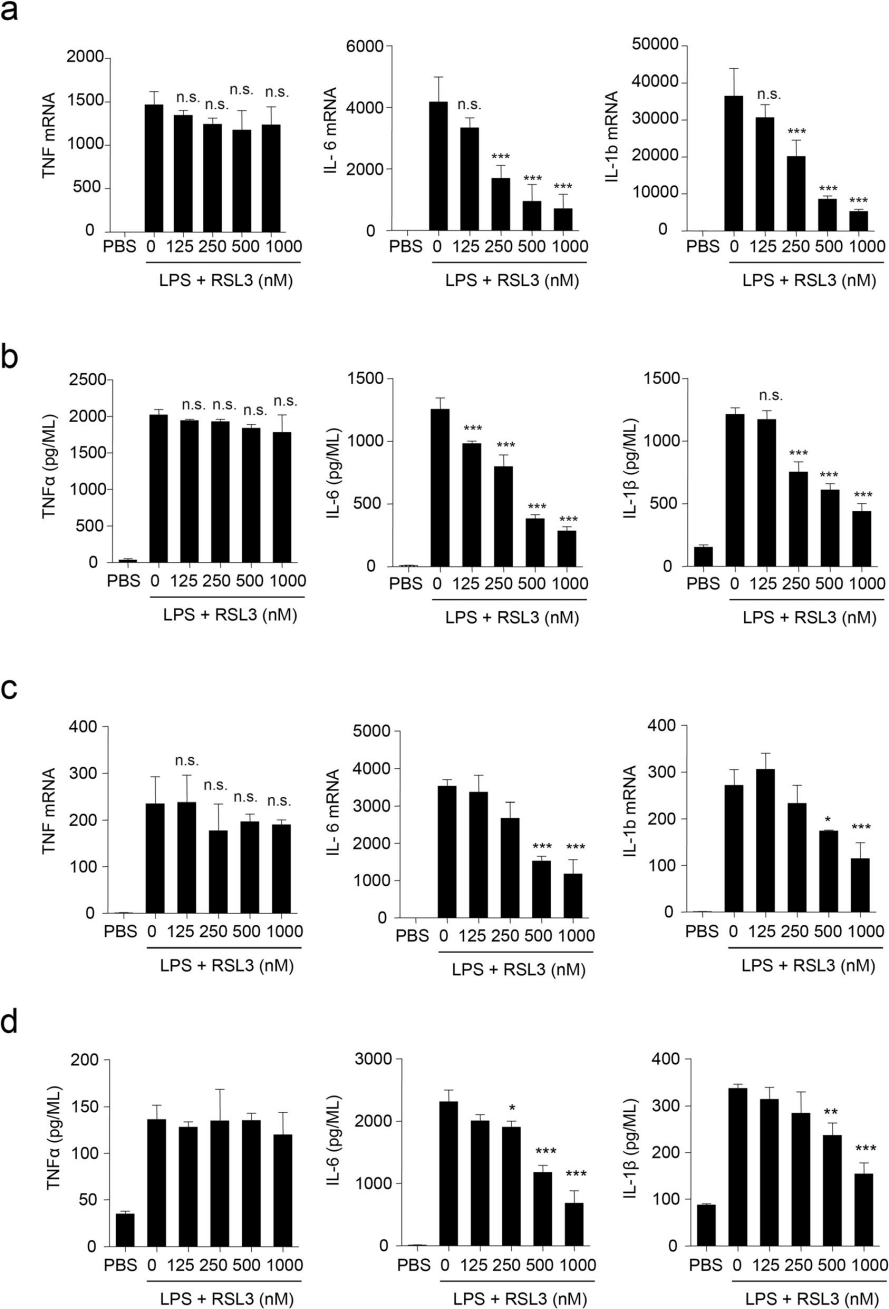
RSL3 inhibits LPS-induced proinflammatory cytokine production of microglia and PMs. **a** qRT-PCR analysis of *TNF*, *IL-6,* and *IL-1b* mRNA levels in LPS (100 ng/ML)-stimulated PMs subjected to different doses of RSL3 treatment for 4 h. **b** ELISA of TNFα, IL-6, and IL-1β in supernatants of LPS-stimulated PMs subjected to different doses of RSL3 treatment for 8 h. **c** qRT-PCR analysis of *TNF*, *IL-6,* and *IL-1b* mRNA levels in LPS (100 ng/ML)-stimulated BV2 cells subjected to different doses of RSL3 treatment for 4 h. **d** ELISA of TNFα, IL-6, and IL-1β in supernatants of LPS-stimulated BV2 cells subjected to different doses of RSL3 treatment for 8 h. The data are means ± SD; for all panels, *P < 0.05, **P < 0.01, ***P < 0.001 by one-way-ANOVA analysis followed by Dunnett test. n.s., no significant. The data are combined from three independent experiments

As cell death has been reported to be tightly related to inflammation, we explored whether ferroptosis could induce inflammation. We first evaluated the consequence of RSL3 treatment on the mRNA level of proinflammatory cytokines including TNFα, IL-6, and IL-1β in PMs following LPS stimulation. Unexpectedly, RSL3 markedly suppressed *IL-6* and *IL-1b* mRNA level in a dose-dependent manner, whereas the abundance of *TNF* was not obviously altered (Fig. 1a). Consistent with the mRNA level, the release of TNFα, IL-6 and IL-1β was also decreased in a dose-dependent manner in PMs upon LPS stimulation after treatment with RSL3 based on ELISA results (Fig. 1b). Thus, RSL3 inhibited LPS-induced inflammatory cytokine production of PMs.

As microglia are tissue-resident macrophages in the central nervous system (CNS), which are critical effectors and regulators in neuroinflammation during development and disease progression [35], we also tested the role of RSL3 in inflammation of microglial cells. Consistent with PMs, primarily cultured microglia (Fig. 1c, d) and BV2 microglial cells (Figure S1a) also exhibited decreased proinflammatory cytokine production upon LPS stimulation in the presence of RSL3. These results indicated that RSL3 suppressed LPS-induced proinflammatory cytokine production.

### Cell ferroptosis induces proinflammatory cytokine production

**Fig. 2:**
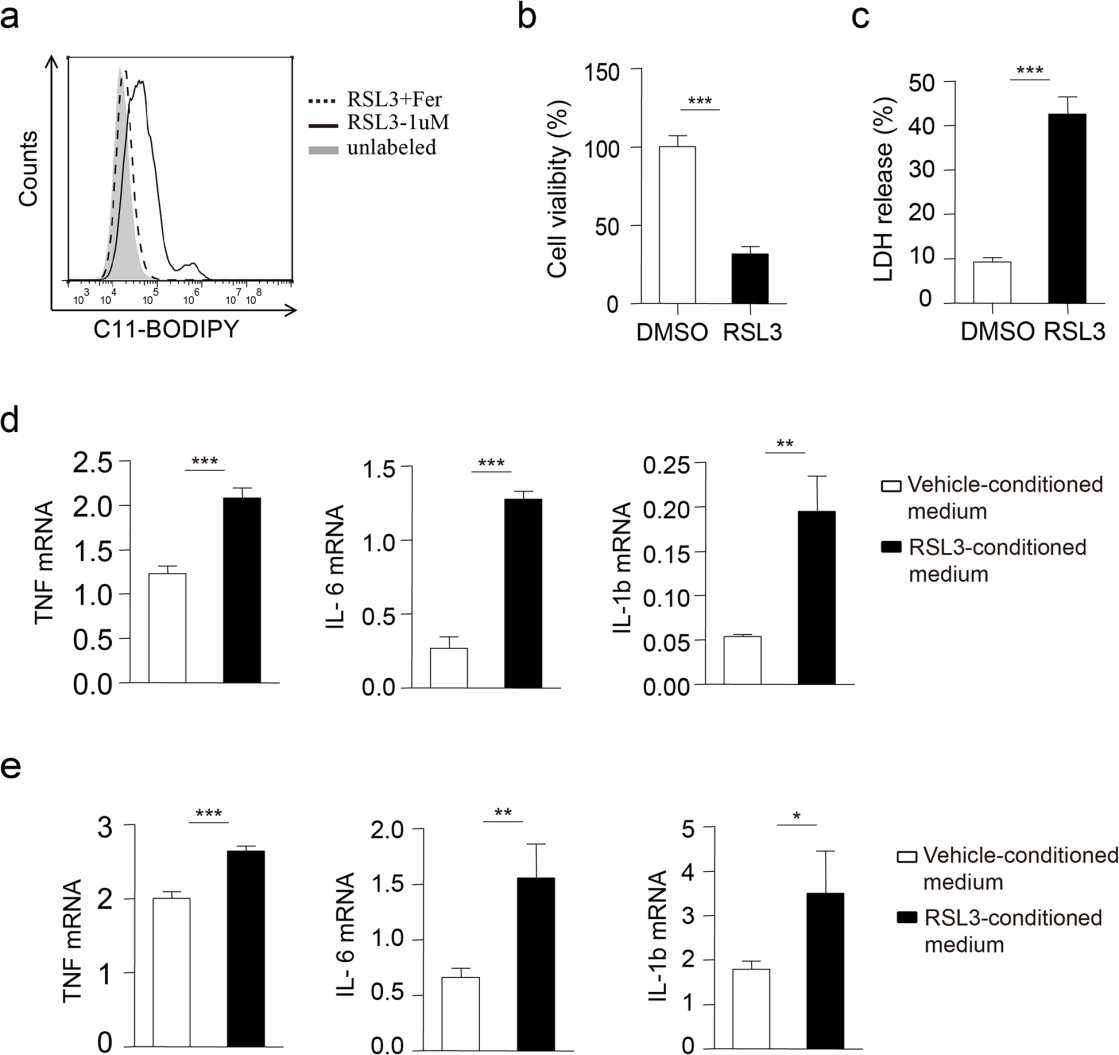
Cell ferroptosis-conditioned medium induces proinflammatory cytokine production. **a** Flow cytometry analysis of C11-BODIPY oxidation in PC12 cells treated with RSL3 (1 μM) or RSL3 plus Fer (1 μM) for 10 h. **b** Cell viability or **c** LDH release was assessed and quantified for PC12 cells subjected to RSL3 (1 μM) treatment for 10 h. qRT-PCR analysis of TNF, IL-6, and IL-1b mRNA levels in primary microglia (**d**) or PMs (**e**) incubated with DMSO-treated cell culture supernatant or RSL3-treated cell culture supernatant for 8 h from PC12 cells. The data are means ± SD; for all panels, *P < 0.05, **P < 0.01, ***P < 0.001 by Student’s *t* test. The data are combined from three independent experiments unless otherwise indicated

To determine whether RSL3-induced ferroptosis repressed inflammatory cytokine production of microglia and PMs, we first used RSL3 to induce ferroptosis of PC12 cells, a rat pheochromocytoma cell line. RSL3 treatment led to obvious lipid peroxidation of PC12 cells (Fig. 2a, Figure S2a-b) based on C11-BODIPY staining, ROS labeling and MDA content test results, and the corresponding cell death (Fig. 2b, c). We then added cell ferroptosis-conditioned medium to culture microglia and measured proinflammatory cytokine production. In contrast to vehicle-conditioned medium-treated microglia, the mRNA level of proinflammatory cytokines including *TNF*, *IL-6,* and *IL-1b* was obviously enhanced in primarily cultured microglia incubated with ferroptosis-conditioned medium (Fig. 2d). In addition, the induced expression of proinflammatory cytokine was also observed in PMs (Fig. 2e). These data suggested that cell ferroptosis-released substance might facilitate proinflammatory cytokine production.

### BV2 microglia and PMs are resistant to ferroptosis

**Fig. 3:**
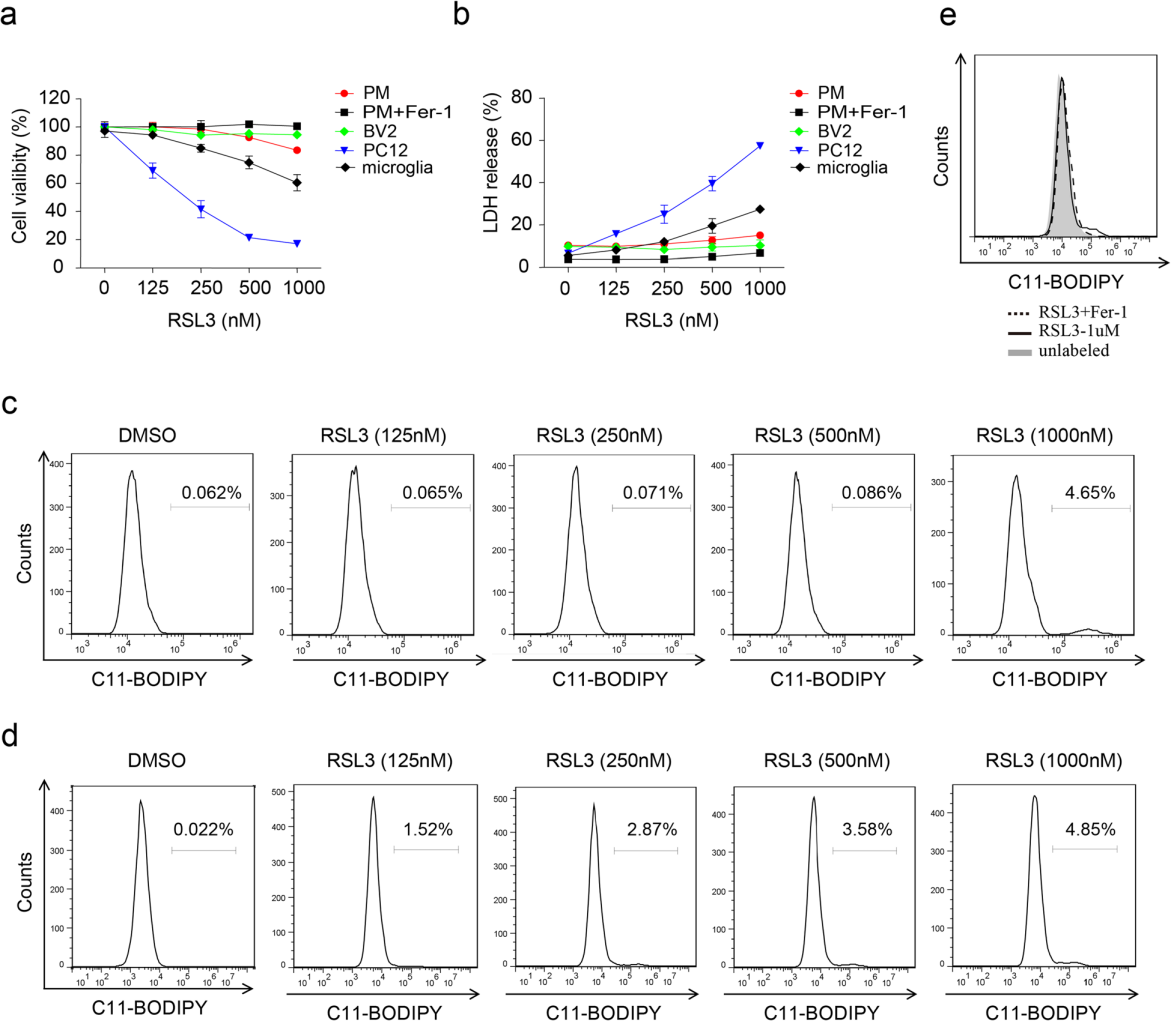
The sensitivity of microglia and macrophages to RSL3-induced ferroptosis. **a** Cell viability of primary microglia, BV2 cells, PMs, and PC12 cells subjected to different doses of RSL3 treatment for 10 h. **b** LDH release assay of microglia, BV2 cells, PMs, and PC12 cells subjected to different doses of RSL3 treatment for 10 h. **c** Flow cytometry analysis of C11-BODIPY oxidation in BV2 cells treated with different doses of RSL3 for 9 h. **d** Flow cytometry analysis of C11-BODIPY oxidation in PMs treated with different doses of RSL3 for 10 h. **e** Flow cytometry analysis of C11-BODIPY oxidation in BV2 cells treated with RSL3 (1 μM) or RSL3 plus Fer-1 (1 μM) for 10 h. The data are means ± SD. All data are representative of or combined from at least three independent experiments

The contradiction of RSL3-inhibited inflammation and cell ferroptosis-induced inflammation intrigued us to investigate whether RSL3-mediated inhibition of inflammation relays on ferroptosis. We first evaluated whether RSL3 could result in ferroptosis of microglia and PMs. CCK8 assay showed that BV2 microglia and PMs were very resistant to RSL3 treatment, primarily cultured microglia were less sensitive to RSL3-induced ferroptosis than PC12 cells (Fig. 3a). Consistently, LDH release assay confirmed the low sensitivity of BV2 microglia and PMs to RSL3 treatment (Fig. 3b). In addition, lipid peroxidation, intracellular ROS and the final product of lipid peroxidation MDA were only slightly accumulated after high dose of RSL3 treatment which could be eliminated by ferroptosis inhibitor Fer-1 (Fig. 3c, e, Figure S2c-f). Therefore, these data demonstrated that BV2 microglia and PMs were resistant to ferroptosis.

We then explored whether other types of macrophages were also showed low sensitivity to RSL3-induced ferroptosis. Conversely, bone marrow-derived macrophages (BMDMs) and RAW264.7 cells were very sensitive to RSL3-induced ferroptosis (Figure S3a-b), compared with PMs and BV2 microglia. These data indicated that macrophages were selectively resistant to RSL3-induced ferroptosis.

### RSL3 suppressed inflammation in a cell ferroptosis-independent manner

**Fig. 4:**
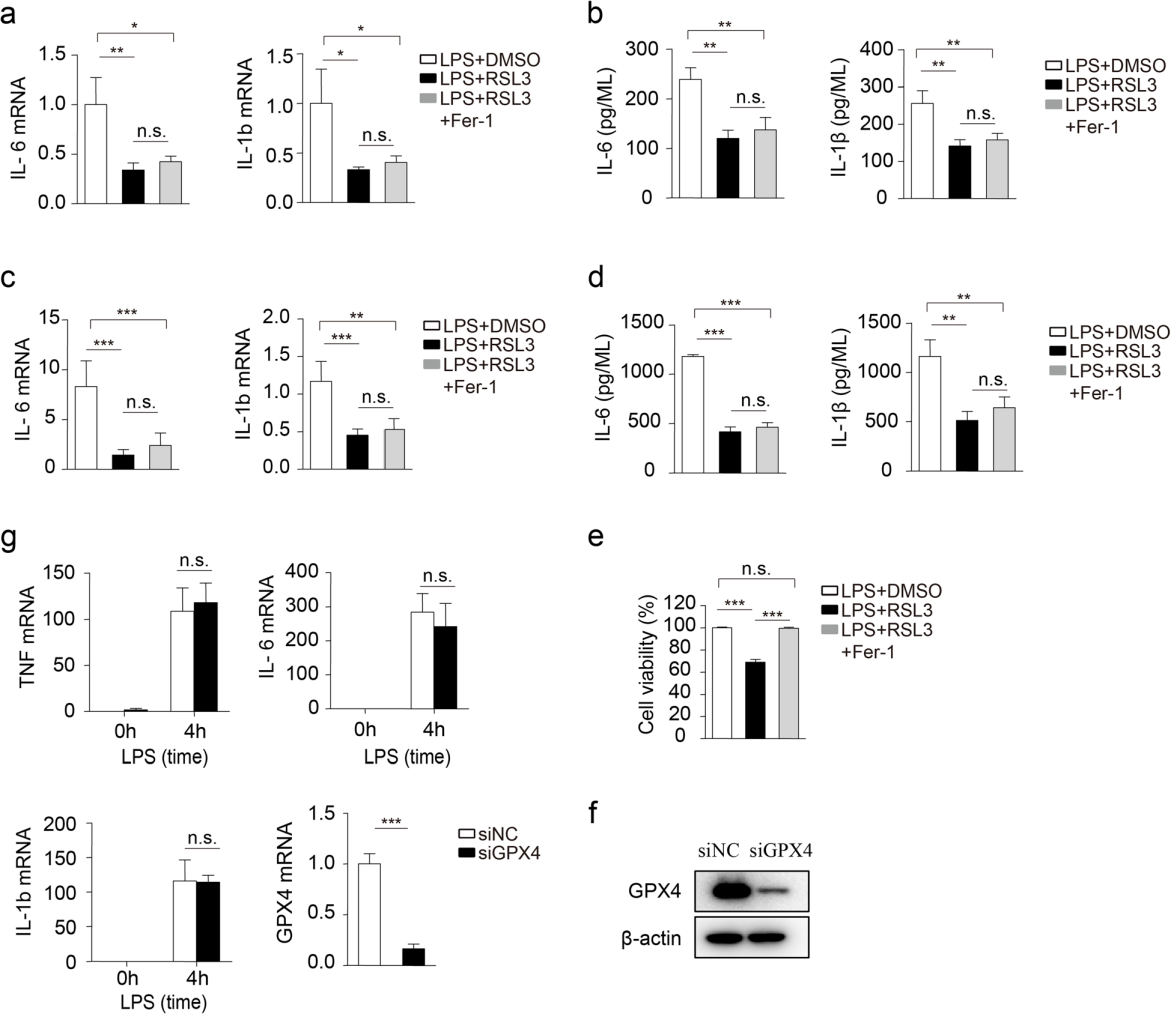
RSL3 inhibits LPS-induced inflammation in a ferroptosis-independent manner. **a** The qRT-PCR analysis of IL-6 and IL-1b mRNA levels in LPS-stimulated microglia subjected to RSL3 (500 nM), RSL3 plus Fer-1 (1 μM) treatment for 4 h. **b** ELISA of IL-6 and IL-1β in supernatants of LPS (100 ng/ML) stimulated microglia subjected to RSL3, RSL3 plus Fer treatment for 8 h. **c** The qRT-PCR analysis of IL-6 and IL-1b mRNA levels in LPS-stimulated PMs subjected to RSL3, RSL3 plus Fer treatment for 4 h. **d** ELISA of IL-6 and IL-1β in supernatants of PMs subjected to RSL3, RSL3 plus Fer treatment for 8 h loading control. **e** Cell viability of primary microglia subjected to RSL3 (1 μM), RSL3 plus Fer treatment in the presence of LPS for 10 h. **f** Representative immunoblot analysis of GPX4 in BV2 microglia transfected with siNC or siGPX4 for 48 h. **g** The qRT-PCR analysis of TNF, IL-6, and IL-1b mRNA levels in LPS-stimulated BV2 microglia transfected with siNC or siGPX4 for 48 h and stimulated with LPS for 4 h. The data are means ± SD, for all panels: *P < 0.05, **P < 0.01, ***P < 0.001. n.s., no significant. **a**–**e** One-way-ANOVA analysis was performed. **g** Two-way-ANOVA analysis followed by Bonferroni post hoc test was performed. All data are representative of or combined from at least three independent experiments

Since RSL3 could induce a low level of lipid peroxidation on BV2 and PMs, we tested whether the suppression of inflammation by RSL3 depended on the slightly induced ferroptosis. Notably, Fer-1, the well-demonstrated ferroptosis inhibitor [36], failed to restore the proinflammatory cytokine production inhibited by RSL3 both mRNA and protein in microglia (Fig. 4a, b) as well as PMs (Fig. 4c, d), although Fer-1 rescued their cell viability diminished by RSL3 (Fig. 4e).

Previous studies showed that RSL3 targets GPX4, the critical regulator of lipid peroxidation to induce cell ferroptosis [10]. We further investigated whether RSL3 inhibited inflammation via GPX4. Knockdown of GPX4 through siRNA-mediated interference was unable to repress inflammatory cytokine production in BV2 microglia (Fig. 4f, g). Collectively, these data indicated that RSL3 repressed inflammation in a cell ferroptosis-independent manner.

### RSL3 inhibits cytokine gene transcription

**Fig. 5:**
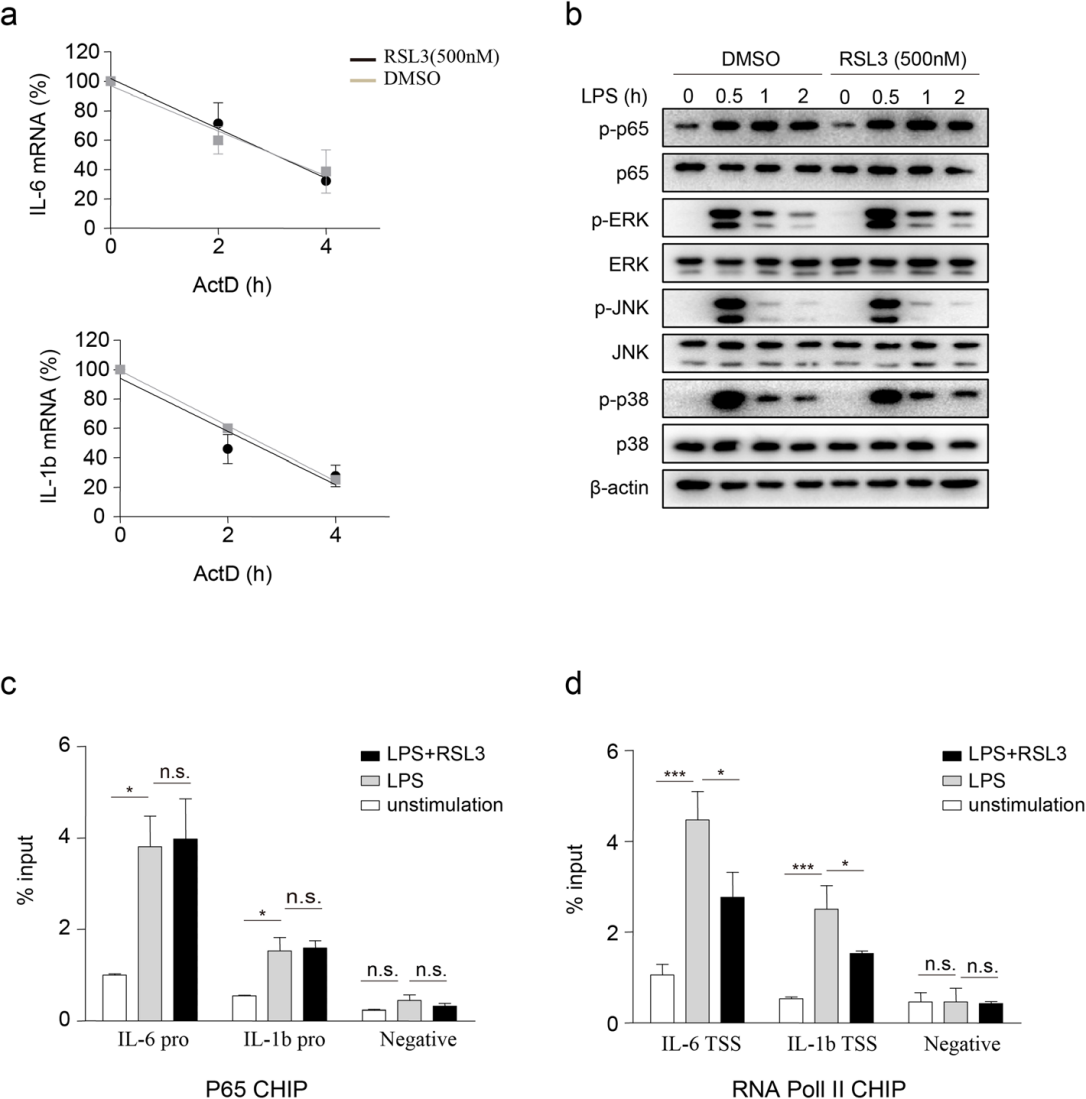
RSL3 inhibits cytokine gene transcription. **a** The qRT-PCR analysis of IL6 and IL-1b mRNA levels in RSL3 or vehicle-treated BV2 microglia subjected to ActD (1 μg/ml) treatment for the indicated times after LPS pre-treated for 2 h. **b** Representative immunoblot analysis of the phosphorylated (p-) or total proteins in lysates of BV2 microglia subjected to RSL3 or DMSO in the presence of LPS (100 ng/ML) for indicated times. ChIP-qRT-PCR analysis of P65 (**c**) and RNA Pol II (**d**) binding in *IL-6* and *IL-1b* loci in BV2 microglia lysates. BV2 microglia were stimulated with LPS (100 ng/ML) or LPS plus RSL3 (500 nM) for 4 h and CHIP assay was then performed. pro, promoter; TSS, transcription start site. The data are means ± SD, *P < 0.05, **P < 0.01, ***P < 0.001 by one-way-ANOVA analysis followed by Turkey test. n.s., no significant. All data are representative of or combined from at least three independent experiments

Given that RSL3 inhibited inflammation in a cell ferroptosis-independent manner, we first tested whether RSL3 could directly promote the degradation of cytokines. After actinomycin D (ActD) treatment, the half-life of *IL-6* and *IL-1b* in LPS-pre-treated BV2 cells (Fig. 5a) and PMs (Figure S4a) was not decreased in RSL3-treated versus DMSO-treated cells. We then examined whether RSL3 regulated the well-established signaling cascades that induced proinflammatory cytokine production after LPS stimulation. Remarkably, the phosphorylation of NF-kB p65, as well as the MAPK kinases ERK, JNK, and p38 was not obviously altered in LPS-triggered BV2 microglia (Fig. 5b) and PMs cells (Figure S4b) in the presence or absence of RSL3. These data indicated that RSL3 might regulate cytokine gene expression by transcriptional inhibition.

To further explore whether RSL3 inhibited cytokine gene transcription, we first used chromatin immunoprecipitation followed by qRT-PCR to test the binding of critical transcription factor P65 to the promoters of *IL-6* and *IL-1b*. However, the recruitment of P65 to the promoters of *IL-6* and *IL-1b* gene was comparable between RSL3-treated BV2 microglia and DMSO-treated BV2 cells (Fig. 5c). We then evaluated whether RSL3 suppressed the recruitment of RNA Pol II to *IL-6* and *IL-1b* transcription start site (TSS), the key step of gene transcription initiation. CHIP assay results showed that RSL3 attenuated the binding of RNA Pol II to the proximity of TSSs of the *IL-6* and *IL-1b* genes in BV2 microglia (Fig. 5d) and PMs (Figure S4c) following LPS stimulation. Therefore, RSL3 might regulate cytokine gene transcription.

### Increased expression of Nrf2 contributes to ferroptosis resistance and inflammation suppression exposed to RSL3

**Fig. 6:**
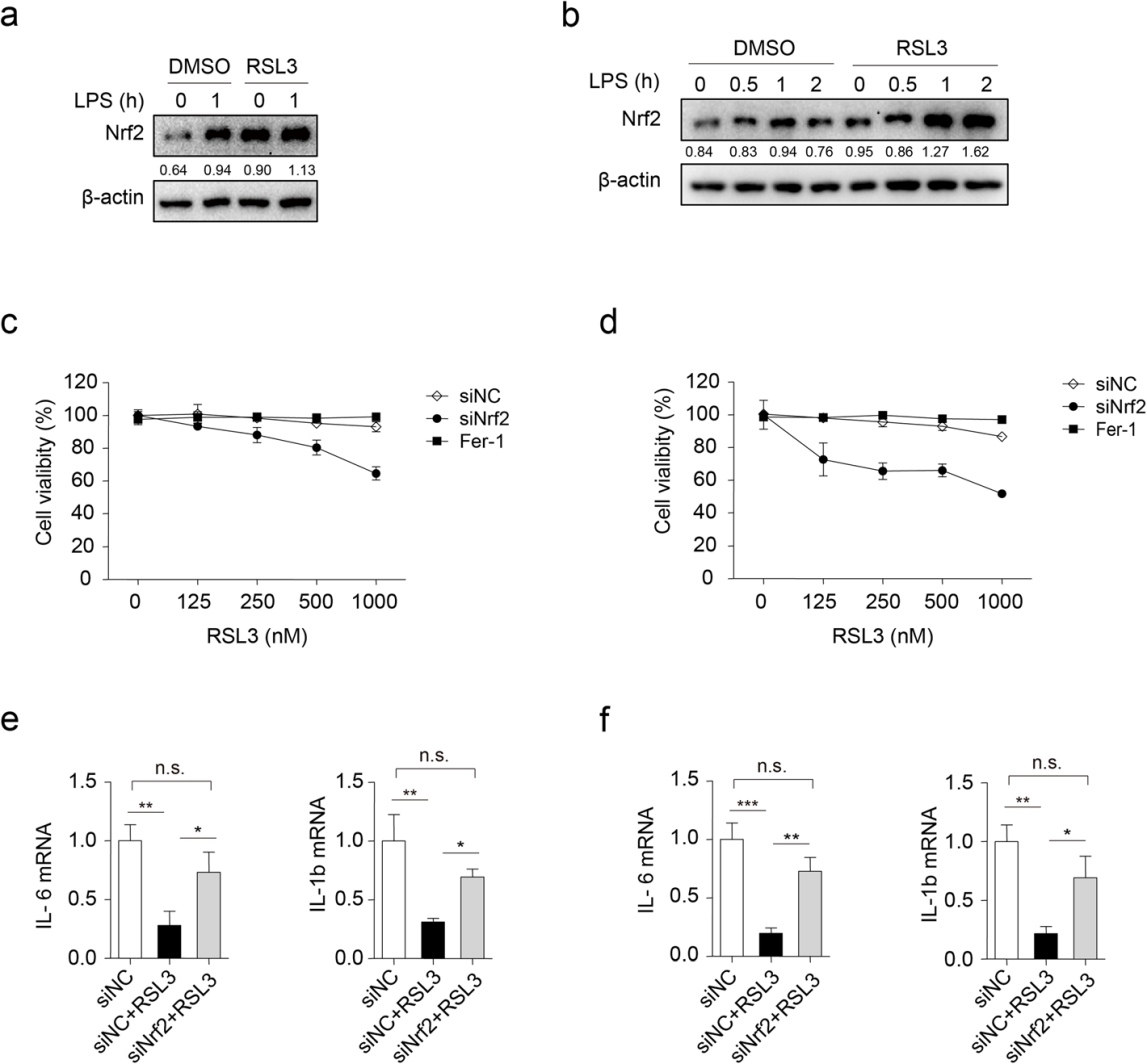
Knockdown of Nrf2 reverses ferroptosis resistance and inflammation suppression in response to RSL3 stimulation. Representative immunoblot analysis of Nrf2 in lysates of BV2 microglia (**a**) or PMs (**b**) subjected to RSL3 (500 nM) or DMSO treatment in the presence of LPS (100 ng/ML) for indicated times. Cell viability of BV2 microglia (**c**) or PMs (**d**) was assessed and quantified transfected with siNC or siNrf2 subjected to different doses of RSL3 treatment for 10 h. The qRT-PCR analysis of *IL-6* and *IL-1b* in BV2 microglia (**e**) or PMs (**f**) transfected with siNC or siNrf2 in the presence or absence of RSL3. One-way-ANOVA analysis was used. The data are means ± SD, *P < 0.05, **P < 0.01, ***P < 0.001 by one-way-ANOVA analysis followed by Turkey test. n.s., no significant. All data are representative of or combined from at least three independent experiments

As the nuclear factor (erythroid-derived 2)-like 2 (Nrf2) has been established to be both anti-inflammatory, involving cytokine transcription repression [37], and anti-oxidation, involving ARE-mediated gene induction [38], we wondered whether RSL3 enhanced Nrf2 expression to suppress cytokine production as well as the sensitivity to ferroptosis. As predicted, the expression of Nrf2 was obviously increased after RSL3 treatment in comparison to DMSO-treated controls both in BV2 microglia (Fig. 6a) and PMs (Fig. 6b). Notably, the mRNA level of Nrf2 was comparable between RSL3-treated PMs and BV2 microglia and vehicle-treated BV2 microglia and PMs (Figure S5a). Thus, RSL3 did not affect Nrf2 expression at the transcriptional level.

We then explored the effects of NRF2 knockdown on cell ferroptosis and inflammation. Knockdown of Nrf2 rendered PMs (Fig. 6c) and BV2 cells (Fig. 6d) more sensitive to RSL3-induced ferroptosis. In addition, Nrf2 knockdown also rescued the reduced mRNA level of IL-6 and IL-1β after RSL3 treatment in BV2 microglia (Fig. 6e, Figure S5b-c) and PMs (Fig. 6f, Figure S5b), which was consistent with the previous report that Nrf2 suppresses inflammatory response by blocking proinflammatory cytokine transcription [37]. Thus, RSL3 increased Nrf2 protein abundance to inhibit inflammation as well as the resistance of BV2 microglia and PMs to ferroptosis.

### RSL3 in combination with ferroptosis inhibitor attenuated LPS-induced inflammation in vivo

**Fig. 7:**
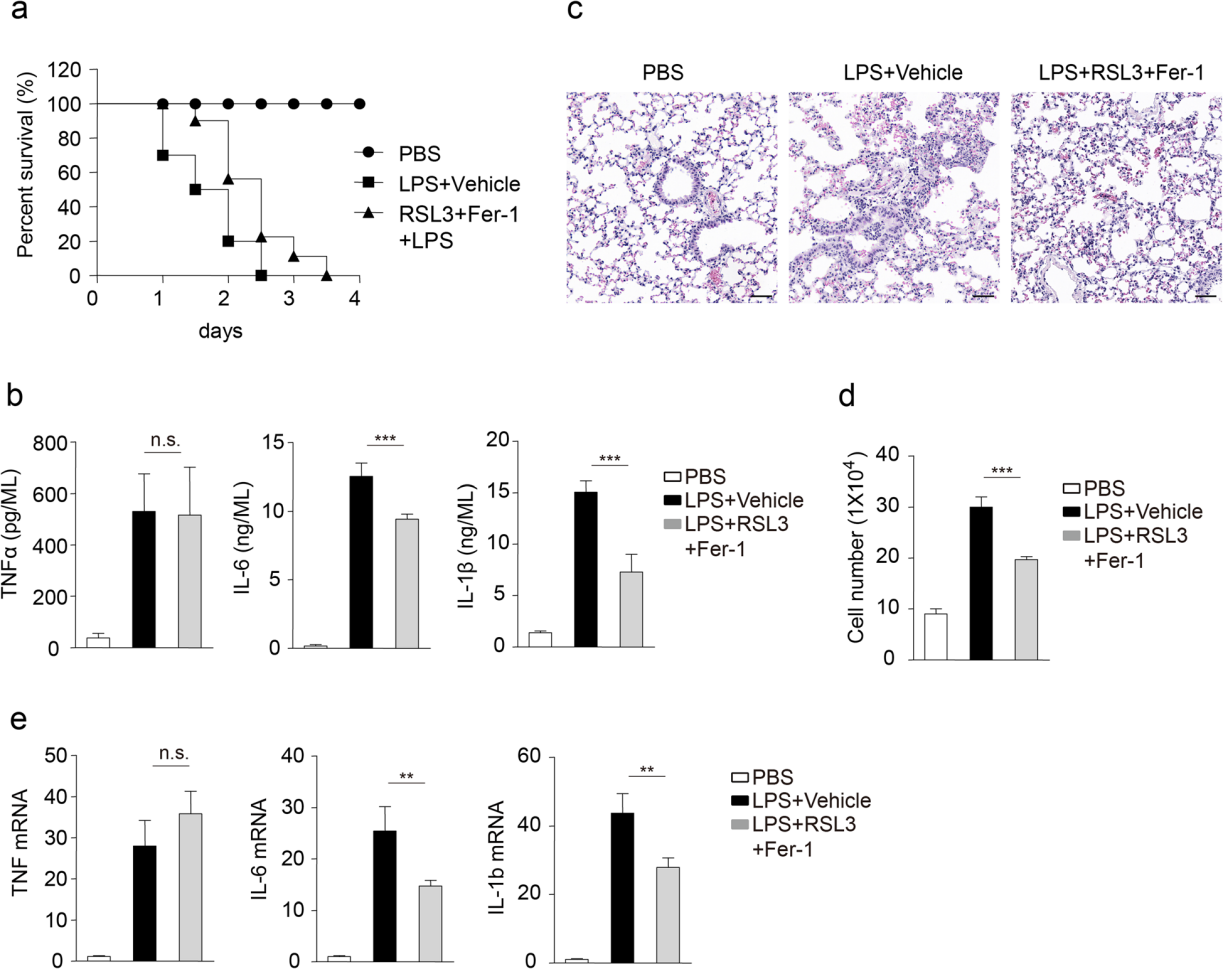
RSL3 in combination with Fer-1 mitigates LPS-induced inflammation. **a** Survival of C57BL/6 mice (injected with RSL3 plus Fer-1 or vehicle; *n* = 9 for each treatment) after lethal challenge with LPS (10 mg/kg body weight). **b** ELISA of TNFα, IL-6, and IL-1β in serum from C57BL/6 mice (injected with RSL3 plus Fer-1 or vehicle; *n* = 5 per group) after challenging with PBS or LPS for 6 h. **c** Hematoxylin and eosin staining of lungs from mice (injected with RSL3 plus Fer-1 or vehicle; *n* = 5 per group) for different treatment after challenging with PBS or LPS. Scale bar, 50 μm. **d** The number of microglia (CD45^int^CD11b^high^) in brain tissues after indicated treatment (LPS, 5 mg/kg body weight) based on flow cytometry analysis. **e** The qRT-PCR analysis of *TNF, IL-6,* and *IL-1b* in brain tissues under different treatment (LPS, 5mg/kg body weight). The data are means ± SD, *P < 0.05, **P < 0.01, ***P < 0.001 by one-way-ANOVA analysis followed by Turkey test. n.s., no significant. All data are representative of or combined from at least three independent experiments

To investigate the importance of RSL3-inhibited inflammation in in vivo conditions, we constructed the systemic LPS model [28, 29]. As some cells are very sensitive to RSL3-triggered ferroptosis, we used RSL3 in combination with ferroptosis inhibitor Fer-1 to reduce the side-effect of RSL3-induced ferroptosis, especially based on the facts that the presence of Fer-1 failed to change the suppressed inflammation raised by RSL3. After lethal challenge with LPS, most mice of vehicle-treated group died within 48 h, whereas mice injected with RSL3 plus Fer-1 exhibited delayed death (Fig. 7a). Consistent with that, we observed less severe infiltration of inflammatory cells in the lungs of RSL3 plus Fer-1-treated mice after LPS challenge than controls injected with vehicle (Fig. 7b). Furthermore, less production of TNFα, IL-6, and IL-1β was observed in serum of mice injected with RSL3 plus Fer-1 than vehicle-injected groups after LPS challenge (Fig. 7c). As systemic LPS injection could also induce neuroinflammation [39], we further investigated the importance of RSL3 plus Fer-1 injection for neuroinflammation after a single sublethal dose injection of LPS. The number of microglial cells was decreased in brains of RSL3 plus Fer-1-treated mice after LPS challenge than controls injected with vehicle (Fig. 7d). In addition, the mRNA abundance of proinflammatory cytokine TNFα, IL-6, and IL-1β in the brain was also decreased after injection with RSL3 plus Fer-1 (Fig. 7e). Thus, these data demonstrated that RLS3 plus Fer-1 treatment mitigated LPS-induced inflammation in in vivo conditions.

## Discussion

As a recently discovered form of cell death, the role of ferroptosis and its inducers in neuroinflammation are unclear. Microglia act as the first responders to infection and injury in the brain. At present, the sensitivity of microglia and other macrophages to ferroptosis inducers are not fully understood. In this study, we showed that ferroptosis inducer RSL3 suppressed LPS-induced inflammation, although ferroptosis-conditioned medium potentiates inflammation. In addition, PMs and BV2 cells were resistant to ferroptosis, whereas BMDMs and RAW264.7 cells were very sensitive to RSL3-induced ferroptosis. Further analysis showed that Nrf2 expression was increased in response to RSL3 treatment to suppress inflammatory cytokine production and increase ferroptosis resistance.

Multiple studies have revealed the tight relationship between cell death and neuroinflammation. On one hand, certain kinds of cell death can lead to neuroinflammation; on the other hand, neuroinflammation can modulate cell death [40, 41]. As a recently discovered way of cell death, the role of ferroptosis in inflammation especially in neuroinflammation is still in its infancy [42]. Previous in vivo studies have shown that GPX4 conditional deletion in kidney or brain leads to the detection of activated astrocytes, microglia, or macrophages [43–46]. In addition, in folic acid-induced acute kidney injury (AKI) models, IL-33 level is increased, and Fer-1 decreases circulating IL-33 levels in AKI [47]. These data may demonstrate the correlation but not direct regulation between ferroptosis and inflammation due to the complex environment in in vivo conditions. Our study, by using cell culture medium from ferroptotic cells to stimulate proinflammatory cytokine production in BV2 cells, directly demonstrates that cells undergo ferroptosis may release some substance that leads to neuroinflammation, although the detailed molecules released and the downstream signaling pathway still need further investigation.

Till now, some ferroptosis inducers and inhibitors have been reported to regulate inflammation in a cell ferroptosis-dependent manner, with the opinion that the pro-ferroptotic agents can trigger inflammation and the anti-ferroptotic agents exert anti-inflammatory effects [48]. In this study, we revealed for the first time that the effects of RSL3 in inhibiting LPS-induced inflammation does not dependent on cell ferroptosis, as Fer-1 is unable to restore the suppressed inflammatory cytokine production. Given that RSL3 can cause cell ferroptosis after intraperitoneal injection, we injected RSL3 plus Fer-1 to restrict inflammation in in vivo conditions, a life-threatening condition which can lead to tissue damage, organ failure, and death [49]. Consistent with our study, erastin, a system x_c_^-^ inhibitor, which limits cystine import and causes GSH depletion [36], has been reported to attenuate septic shock and inflammatory gene expression through suppressing the NF-κB pathway, although the author did not mention whether erastin-mediated inhibition of inflammation relies on ferroptosis [50]. Considering the obvious inhibition of inflammation by RSL3, it will be interesting to design a modified anti-neuroinflammation drug based on RSL3 structure while avoiding its role of inducing ferroptosis.

Recently, ferroptosis inducers have been raised as a novel therapeutic approach for the treatment of cancer, such as glioma and renal cell carcinoma [51], as many cancer cells are sensitive to RSL3 or other inducers-induced ferroptosis. Based on our current results, we wonder whether the obvious effect of RSL3 on cancer killing is partly caused by RSL3-inhibited inflammation in local conditions, as inflammation promotes cancer proliferation and progression [52], although this point still needs further investigation. Therefore, in future ferroptosis-based treatment in diseases, the suppressed inflammatory response should be considered to reach optimal treatment effects.

As ferroptosis participates in various diseases, uncovering how cells resist ferroptosis is necessary for exploiting ways to the treatment of cancer and other diseases. One previous study showed that some cancer cells resist ferroptosis by enabling a PROMININ2-dependent iron export pathway involving exosome trafficking of iron to extracellular spaces, diminishing the intracellular iron needed for ferroptosis [21, 22]. The α6β4 integrin mediates activation of Src and STAT3 to suppress expression of ACSL4, an enzyme that enriches membranes with long polyunsaturated fatty acids and is required for ferroptosis to protect adherent epithelial and carcinoma cells from ferroptosis induced by erastin [23, 24]. Ferroptosis suppressor protein 1 (FSP1), another ferroptosis resistance factor can be recruited to the cell membrane to reduce coenzyme Q10 (CoQ), which acts as a lipophilic radical-trapping anti-oxidant that halts the propagation of lipid peroxides [53]. In this study, we revealed that BV2 microglia and peritoneal macrophages are resistant to ferroptosis, which may result from increased Nrf2 expression, a transcription factor that has been demonstrated to enhance the resistance of cancer cells to ferroptosis [25, 54]. These studies collectively indicate that pathways that are decreasing iron metabolism and lipid peroxidation accumulation, increasing anti-oxidant signaling may confer cells with ferroptosis resistance, and blocking these pathways may provide a way to elevate cell ferroptosis sensitivity and cure certain disease such as cancer.

Nrf2 is a well-established transcription factor that can be both anti-inflammatory and anti-oxidant [38]. Most previous studies showed that Nrf2 inhibits inflammation in an ARE-dependent manner [55], which is also the mechanism of ROS elimination. Notably, one previous study showed that Nrf2 can inhibit LPS-induced inflammation by blocking proinflammatory cytokine transcription, which is independent of their ROS elimination effect [37]. In our study, we observed obvious inhibition of inflammatory cytokine transcription by RSL3, which fails to be rescued by Fer-1, the scavenger of lipid radicals [56]. Consistent with the previous study, knockdown of Nrf2 rescues the suppressed inflammatory cytokine transcription in an ARE-independent manner, although the reduced expression also rendered cells more sensitive to ferroptosis, which may rely on ARE-mediated anti-oxidant gene transcription.

## Conclusion

In the present study, we showed the proinflammatory role of ferroptosis in microglia and macrophages, revealed RSL3 as a potent neuroinflammation inhibitor, and uncovered the mechanism of microglia and macrophage resistance to ferroptosis. To obtain optimal effects, targeting ferroptosis in diseases by using RSL3 should both consider the ferroptosis effect and the anti-inflammation effect.

## Supplementary Information

**Additional file 1: Table 1.** Primers for qRT-PCR analysis. Table 2. Primers for CHIP-qRT-PCR analysis [1]. Supplementary Figure legends

**Additional file 2: Figure S1.** RSL3 inhibits proinflammatory cytokine production in BV2 cells

**Additional file 3: Figure S2.** The level of cellular ROS and MDA in different cells

**Additional file 4: Figure S3.** BMDM and RAW264.7 cells are sensitive to RSL3 treatment

**Additional file 5: Figure S4.** RSL3 affects the binding of RNA POL II on TSS of IL-6 and IL-1b

**Additional file 6: Figure S5.** Nrf2 expression after RSL3 treatment and knockdown

**Additional file 7: Figure S6.** RAW data of our Western Blot experiments

## Data Availability

All data generated or analyzed during this study are included in this published article.

## Abbreviations

LPS: Lipopolysaccharides
PMs: Peritoneal macrophages
PRRs: Pattern recognition receptors
PAMPs: Pathogen-associated molecular patterns
TLRs: Toll-like receptors
Fer-1: Ferrostatin-1
BMDMs: Bone marrow-derived macrophages
LDH: Lactate dehydrogenase
ATCC: American Type Culture Collection
FBS: Fetal bovine serum
CHIP: Chromatin immunoprecipitation assay
CNS: Central nervous system
ActD: Actinomycin D
TSS: Transcription start site
Nrf2: Nuclear factor (erythroid-derived 2)-like 2
AKI: Acute kidney injury
ROS: Reactive oxygen species
MDA: Malondialdehyde
