# Supplementary material for: Microglia and macrophage exhibit attenuated inflammatory response and ferroptosis resistance after RSL3 stimulation via increasing Nrf2 expression
Source: J Neuroinflammation. 2021 Oct 30;18:249. doi: 10.1186/s12974-021-02231-x (PMC8557003; doi:10.1186/s12974-021-02231-x)
Supplement: Supplementary file 1 — Additional file 1: Table 1. Primers for qRT-PCR analysis. Table 2. Primers for CHIP-qRT-PCR analysis [1]. Supplementary Figure legends [file 12974_2021_2231_MOESM1_ESM.docx]

**Microglia and Macrophage Exhibit Attenuated Inflammatory Response and Ferroptosis Resistance after RSL3 Stimulation via Increasing Nrf2 Expression**

Yu Cui^1^, Xin Zhou^1^, Zhiyuan Zhao^2^, Rui Zhao^2^, Xiangyu Xu^2^, Xiangyi Kong^1^, Jinyang Ren^1^, Xujin Yao^1^, Zhaolong Zhang^2^, Qian Wen^3^, Feifei Guo^4^, Shengli Gao^4^, Jiangdong Sun^1^ and Qi Wan^1*^

^1^Institute of Neuroregeneration & Neurorehabilitation, Department of Pathophysiology,

Qingdao University, Ningxia road 308, Qingdao 266071, China

^2^The Affiliated Hospital of Qingdao University, Jiangsu Road 16, Qingdao 266000, Shandong, China

^3^Department of Biomedical Center, Qingdao University, Qingdao, 266071, China

^4^School of Basic Medicine, Qingdao University, Ningxia road 308, Qingdao 266071, China

* Correspondence should be addressed to: Dr. Qi Wan, Institute of Neuroregeneration & Neurorehabilitation, Qingdao University, 308 Ningxia Street, Qingdao 266071, China. E-mail address: qiwan1@hotmail.com

**Supplemental Tables**

**Table1. Primers for qRT-PCR analysis**

| **Name** | **Sequence** |
| --- | --- |
| TNF-RT-FP | TGATCGGTCCCCAAAGGGATG |
| TNF-RT-RP | TTGGTGGTTT GCTACGACGTGG |
| IL-6-RT-FP | TGATGCACTTGCAG AAAACAATCTGA |
| IL-6-RT-RP | AGCTATGGTACTCCAGAAGACCA GAGG |
| IL1b-RT-FP | GCAACTGTTCCTGAACTCAACT |
| IL1b-RT-RP | ATCTTTTGGGGTCCGTCAACT |
| Nrf2-RT-FP | GTAGATGACCATGAGTCGCTTGCC |
| Nrf2-RT-RP | AGTCATGGCTGCCTCCAGAGAGC |
| Gapdh-RT-FP | TGGTGAAGGTCGGTGTGAACGG |
| Gapdh-RT-RP | ACTGTGCCGTTGAATTTGCCG |

**Table 2. Primers for CHIP-qRT-PCR analysis[1].**

| **Name** | **Sequence** |
| --- | --- |
| IL-6-pro-FP | TCCCATCAAGACATGCTCAA |
| IL-6-pro-RP | AGGAAGGGGAAAGTGTGCTT |
| IL-6-TSS-FP | CCGGAGAGGAGACTTCACAG |
| IL-6-TSS-RP | TCCACGATTTCCCAGAGAAC |
| IL1b-pro-FP | GGGAGAAGCTTGATGGGAAT |
| IL1b-pro-RP | TATCTGCCACCCCTTGACTT |
| IL1b-TSS-FP | AGATGCTCTGGAAGGAAGCA |
| IL1b-TSS-RP | GGCAGCTCCTGTCTTGTAGG |
| Negative-FP | GAAGATGCCTTCAAAGGACAAGTAC |
| Negative-RP | ATGACAGGTCCAAACGAGAG |

**Supplementary Figure legends**

**Figure S1. RSL3 inhibits proinflammatory cytokine production in BV2 cells.**

(a)qRT-PCR analysis of *TNF*, *IL-6* and *IL-1b* mRNA levels in LPS (100 ng/ML)-stimulated BV2 cells subjected to RSL3 (500nM) treatment for 4 h. The data are means ± S.D., for all panels: *P < 0.05, **P < 0.01, ***P < 0.01 by one-way ANOVA analysis. All data are combined from three independent experiments.

**Figure S2. The level of cellular ROS and MDA in different cells.**

(a) Flow-cytometric analysis of ROS level based on DCFH-DA dye staining in PC12 cells-treated with DMSO or RSL3 (1μM) for 10 h. (b-c) Quantification of mean fluorescence intensity (MFI) of intracellular ROS level by DCFH-DA staining of BV2 cells (b) or PMs (c) in the presence of different doses of RSL3 for 10 h. (d) The content of MDA in PC12 cells-treated with DMSO or RSL3 (1μM) for 10 h. (e-f) The content of MDA in BV2 microglia (e) or PMs (f) in the presence of different doses of RSL3 for 10 h. The data are means ± S.D., *P < 0.05, **P < 0.01, ***P < 0.01 by one-way ANOVA followed by Dunnett test.

**Figure S3. BMDM and RAW264.7 cells are sensitive to RSL3 treatment.**

(a) Cell viability of BMDM or RAW264.7 cells subjected to different doses of RSL3 treatment for 10 h. (b) LDH release of BMDM or RAW264.7 cells subjected to different doses of RSL3 treatment for 10 h. All data are combined from three independent experiments.

**Figure S4. RSL3 affects the binding of RNA POL II on TSS of *IL-6* and *IL-1b*.**

(a) The qRT-PCR analysis of *IL-6* and *IL-1b* mRNA levels in RSL3 or vehicle-treated PMs subjected to ActD (1µg/ml) treatment for the indicated times after LPS pre-treated for 2 h. (b) Representative immunoblot analysis of the phosphorylated (p-) or total proteins in lysates of PMs subjected to RSL3 (500 nM) or DMSO in the presence of LPS (100 ng/ML) for indicated times. (c) ChIP-qRT-PCR analysis of RNA Pol II binding in *IL-6* and *IL-1b* loci in PMs lysates. PMs were stimulated with LPS (100 ng/ML) or LPS plus RSL3 for 4 h and CHIP assay was then performed. TSS means transcription start site. The data are means ± S.D., for all panels: *P < 0.05, **P < 0.01, ***P < 0.01 by one-way ANOVA analysis. The data are representative of or combined from three independent experiments.

**Figure S5.** **Nrf2 expression after RSL3 treatment and knockdown.**

(a)The qRT-PCR analysis of Nrf2 mRNA levels in LPS stimulated PMs (left panel) or BV2 cells (right panel) subjected to RSL3(500 nM) or DMSO treatment for 4 h. (b) The qRT-PCR analysis of Nrf2 mRNA levels in PMs (left panel) or BV2 cells (right panel) transfected with siNC or siNrf2 for 48 h. (c) Representative immunoblot analysis of Nrf2 expression in lysates of BV2 microglia transfected with siNC or siNrf2 and subjected to RSL3 or DMSO treatment in the presence of LPS (100 ng/ML) for indicated times. The data are means ± S.D., for all panels: *P < 0.05, **P < 0.01, ***P < 0.01, by two-way ANOVA analysis for (a) and students’ t test for (b). n.s., no significant. The data are representative of or combined from three independent experiments.

**Figure S6. RAW data of our Western Blot experiments.**

**References**

[1] Kobayashi EH, Suzuki T, Funayama R, Nagashima T, Hayashi M, Sekine H, et al. Nrf2 suppresses macrophage inflammatory response by blocking proinflammatory cytokine transcription**.** Nat Commun 2016;711624.
