# Supplementary figures and images for: Microglia and macrophage exhibit attenuated inflammatory response and ferroptosis resistance after RSL3 stimulation via increasing Nrf2 expression
Source: J Neuroinflammation. 2021 Oct 30;18:249. doi: 10.1186/s12974-021-02231-x (PMC8557003; doi:10.1186/s12974-021-02231-x)

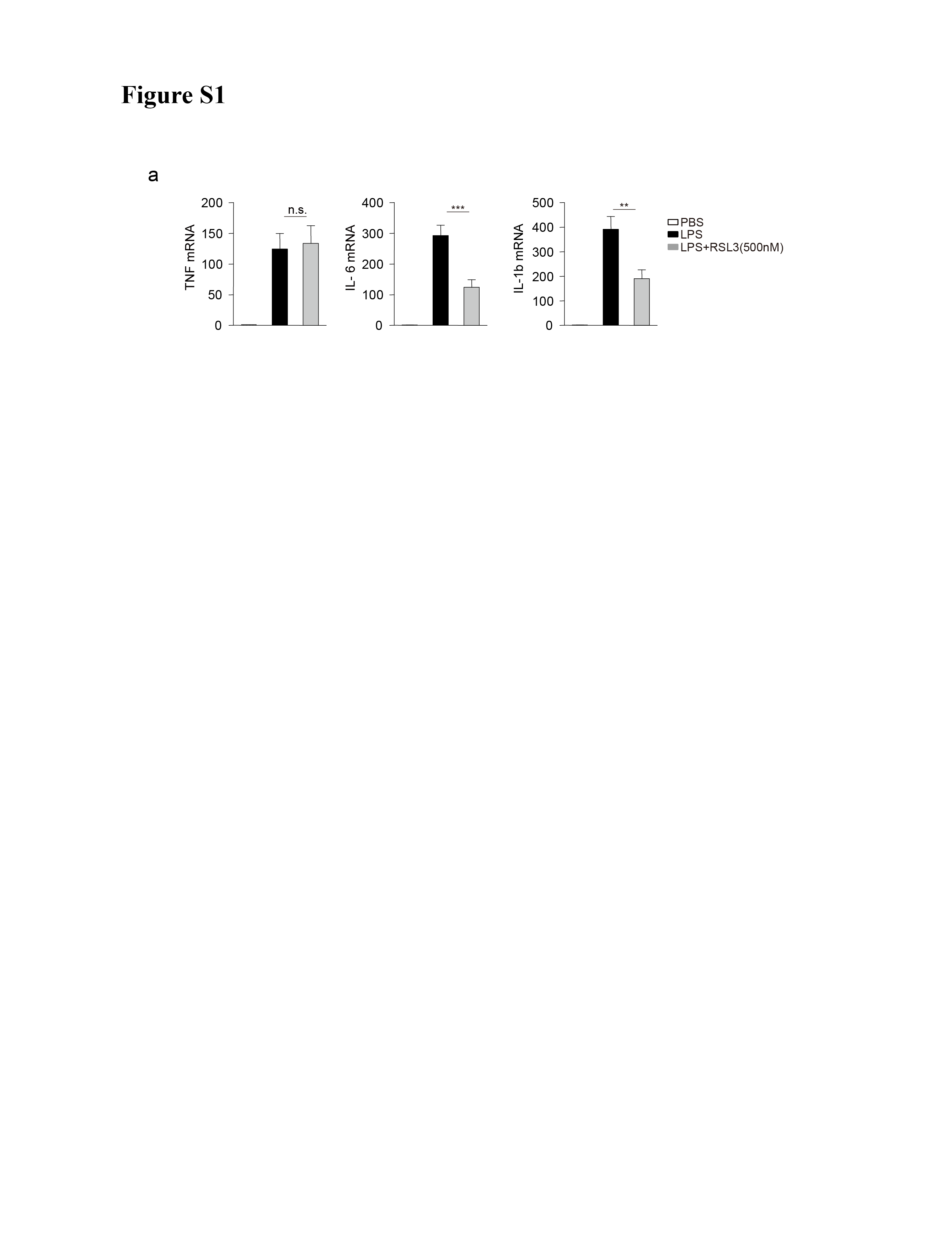

Supplement: Supplementary file 2 — Additional file 2: Figure S1. RSL3 inhibits proinflammatory cytokine production in BV2 cells [file 12974_2021_2231_MOESM2_ESM.tif]

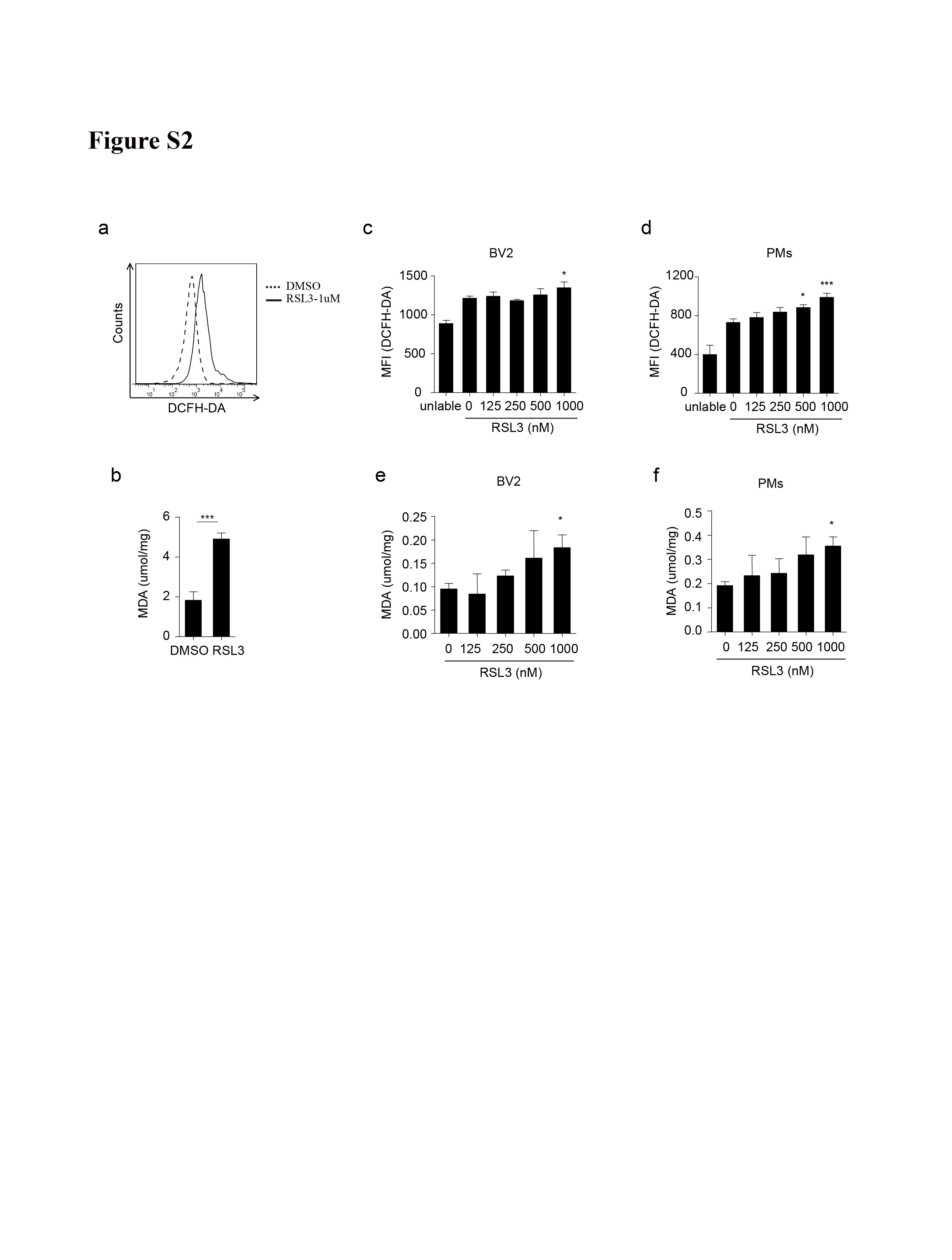

Supplement: Supplementary file 3 — Additional file 3: Figure S2. The level of cellular ROS and MDA in different cells [file 12974_2021_2231_MOESM3_ESM.tif]

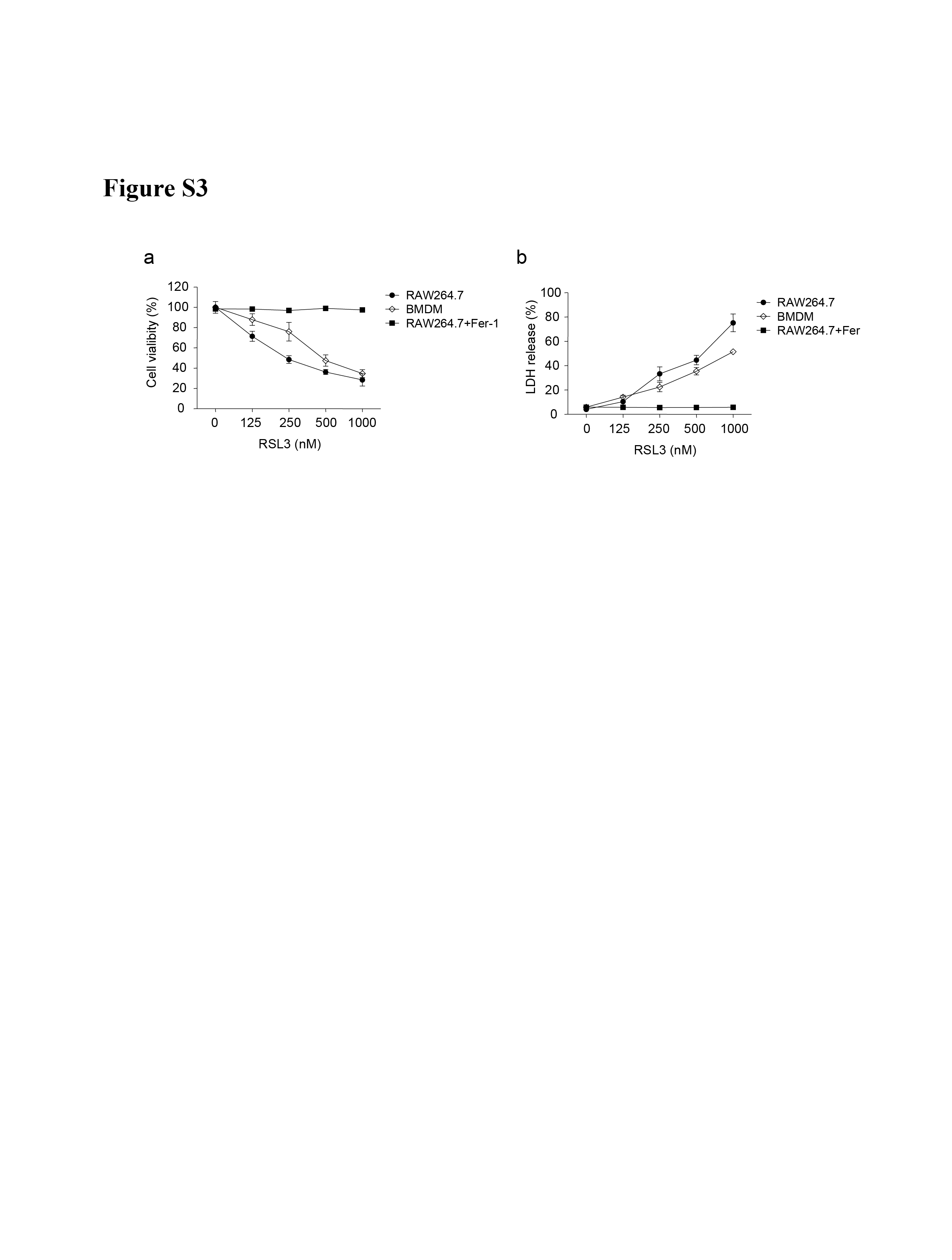

Supplement: Supplementary file 4 — Additional file 4: Figure S3. BMDM and RAW264.7 cells are sensitive to RSL3 treatment [file 12974_2021_2231_MOESM4_ESM.tif]

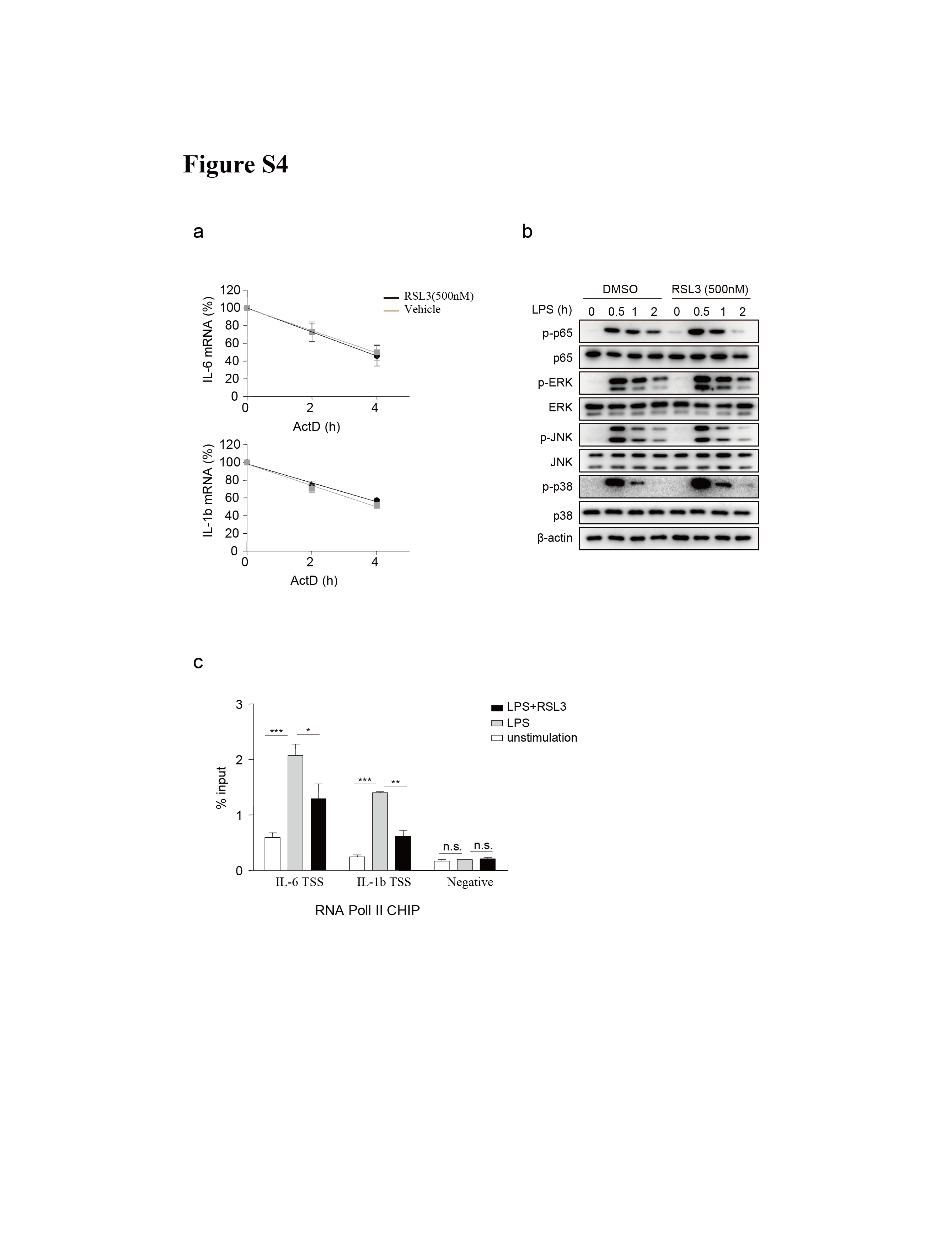

Supplement: Supplementary file 5 — Additional file 5: Figure S4. RSL3 affects the binding of RNA POL II on TSS of IL-6 and IL-1b [file 12974_2021_2231_MOESM5_ESM.tif]

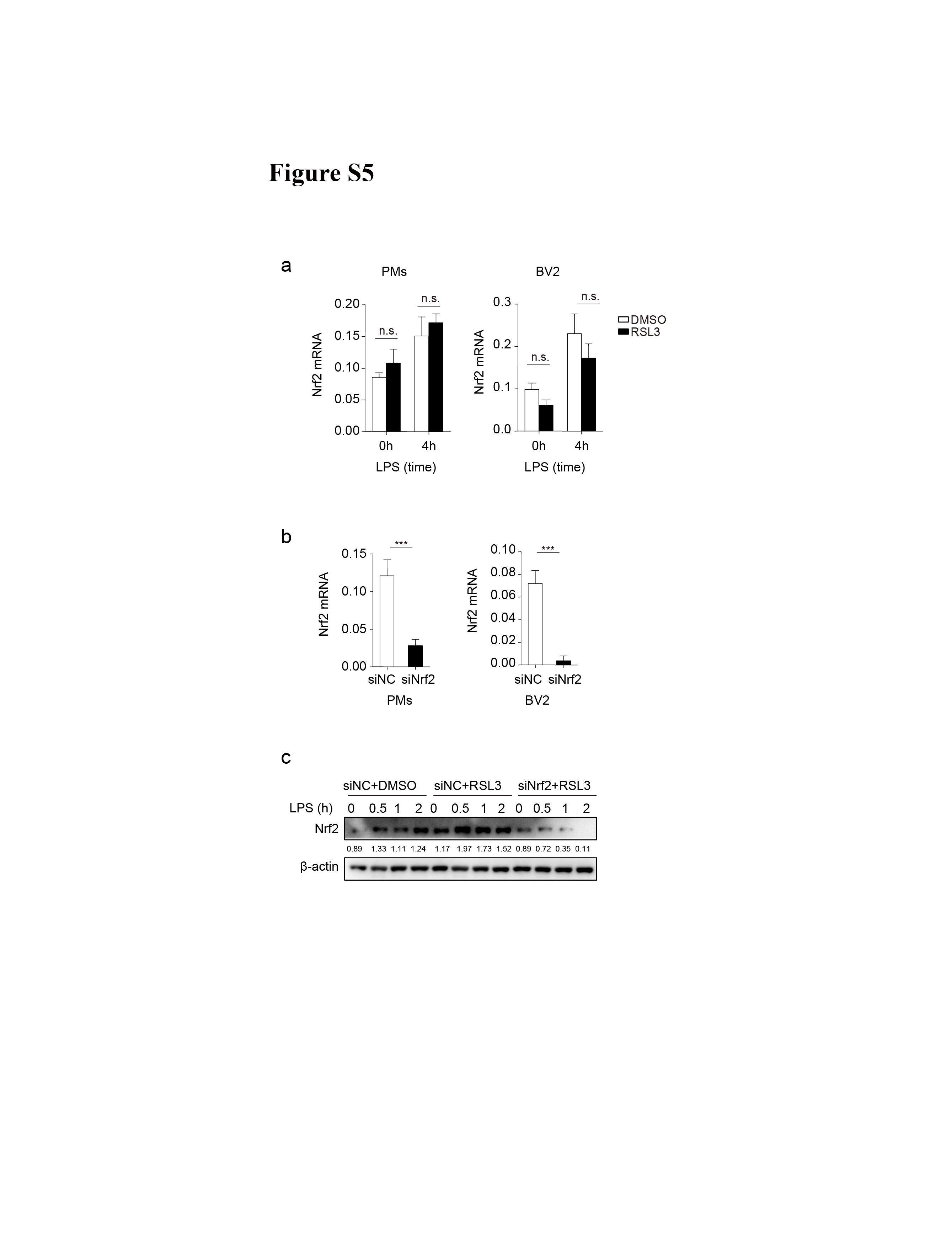

Supplement: Supplementary file 6 — Additional file 6: Figure S5. Nrf2 expression after RSL3 treatment and knockdown [file 12974_2021_2231_MOESM6_ESM.tif]

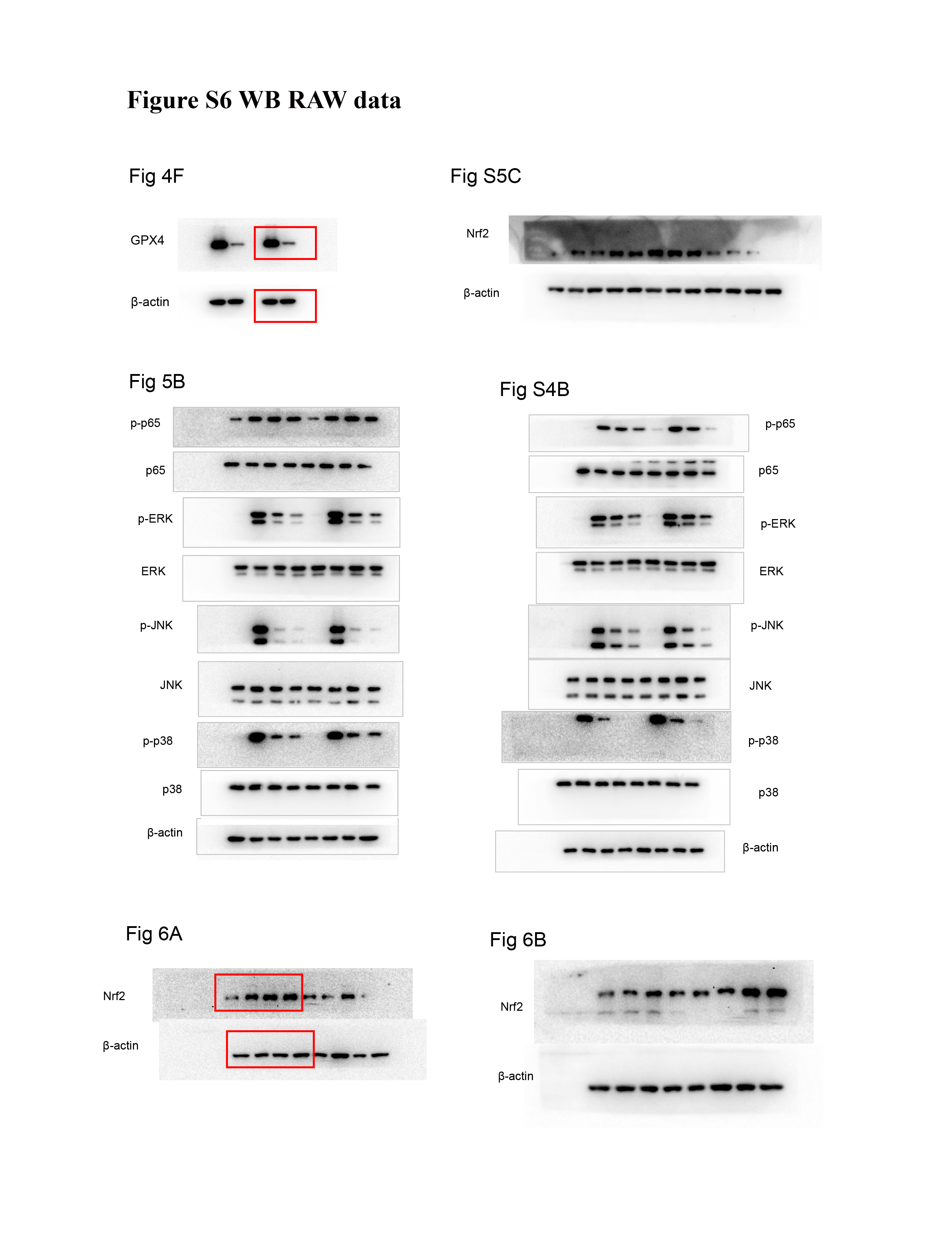

Supplement: Supplementary file 7 — Additional file 7: Figure S6. RAW data of our Western Blot experiments [file 12974_2021_2231_MOESM7_ESM.tif]
